# Evaluation of antibiotic dispensing practice in community pharmacies in Jordan: A cross sectional study

**DOI:** 10.1371/journal.pone.0216115

**Published:** 2019-04-29

**Authors:** Randa N. Haddadin, Mervat Alsous, Mayyada Wazaify, Linda Tahaineh

**Affiliations:** 1 Pharmaceutics and Pharmaceutical Technology Department, School of Pharmacy, The University of Jordan, Amman, Jordan; 2 Department of Pharmacy Practice, School of Pharmacy, Yarmouk University, Irbid, Jordan; 3 Clinical Pharmacy Department, School of Pharmacy, The University of Jordan, Amman, Jordan; 4 Clinical Pharmacy Department, Faculty of Pharmacy, Jordan University of Science and Technology, Irbid, Jordan; Universidade Nova de Lisboa, PORTUGAL

## Abstract

It is well known that the emergence of antibiotic resistance is linked to the misuse and overuse of antibiotics. Misuse includes self-medication and the inappropriate use of antibiotics because of improper dosage or improper duration than recommended. This study investigated three patterns of dispensing antibiotics in a sample of community pharmacies in Jordan. This included dispensing antibiotics by prescription or over-the-counter either by direct request or upon a pharmacist’s recommendation. The antibiotics dispensed were evaluated in terms of indication, appropriateness of dose, and duration of treatment based on the empirical treatment suggested by selected references: Lexicomp (2017) and UptoDate (2017) and the manufacturer’s recommendations. Of the 457 antibiotics dispensed, almost one third were without prescription. Of the antibiotics dispensed with prescription or without prescription, 31.5% and 24.6% respectively were appropriate dosage and duration (*p* = 0.002). In the three patterns of dispensing, beta lactam antibiotics were the most commonly dispensed. In addition, it was noticed that there was a tendency to prescribe or dispense higher generations of antibiotics to cases that could have been treated with lower generation or safer antibiotics. Furthermore, 12.2% of the antibiotics were dispensed to treat infections that are not indicated for them. In conclusion, a significant proportion of antibiotics are dispensed without prescription in Jordan. Moreover, a considerable proportion of prescribed antibiotics were inappropriate for the conditions concerned. This indicates the importance of enforcing the Jordanian regulations prohibiting the dispensing of nonprescription antibiotics and the implementation of continuous education to physicians and pharmacists to increase awareness about the emergence of antibiotic resistance.

## Introduction

Antibiotic resistance is among the biggest threats to global health. With time, it is rising to more dangerous levels worldwide. Two years ago, World health organization (WHO) addressed its concerns about running out of antibiotic options to treat some resistant bacterial infections [1].

It is well known that antibiotic resistance occurs as a natural process at low rates [2]. However, the misuse or irrational use of antibiotics in humans accelerates the emergence and spread of the process [3]. Irrational use includes the use of antibiotics to treat viral infections, negligent use, or the wrong drug or inappropriate dosage or duration for treatment [3–5].

The WHO, in its two reports on ‘Global Strategy for Containment of Antimicrobial Resistance’ and ‘The Pursuit of Responsible Use of Medicines’ [5,6], recognized the irrational use of antibiotics as a major factor in developing antimicrobial resistance that should be confronted by governments.

The resistance occurs due to selective pressure exerted on microbial communities leading to the selection of resistant strains [1]. The irrational use of antibiotics is obvious in self-medication practiced by the public worldwide. Also, it was reported in many countries among healthcare workers through irrational prescription of antibiotics. This drove the WHO to urge the countries to set action plans to tackle these problems [5,6]. Studies have shown that antibiotic self-medication is linked to the lack of legislation that prohibits dispensing antibiotics without prescriptions, poor enforcement of regulations, high cost of medical consultation, and lack of health insurance [4,7]. On the other hand, over prescription or inappropriate prescription of antibiotics was reported among physicians in different hospitals [8]. Among the reasons for this irrational prescription of antibiotics was the reliance of physicians on their past experience or on information from pharmaceutical representatives rather than laboratory diagnosis and regional or national treatment guidelines [6,8].

In Jordan, patients can obtain a large number of medicines, including antibiotics, without prescription, except for narcotics and some controlled drugs [9]. One study in Jordan found that 42.5% of dispensed antibiotics were without prescription, and had been requested by the customer by brand name [10]. Although regulations in Jordan prohibits the dispensing of antibiotics without prescription, such regulations are commonly not enforced [9]. Previous studies in Jordan reported misuse of antibiotics in community pharmacy settings [9,11] and among the general public [12]. However, these studies were based on questionnaires that were distributed to the pharmacies or the general population, in which the outcomes could be subjective.

Therefore, we intended to construct an observational study for this research. In our study we adopted an observational method rather than the more commonly used questionnaire method, since observational methods study real life samples of everyday pharmacy practice. This was considered preferable to the potentially falsifiable and subjective questionnaire approach. In fact, observational studies are increasingly used in the study of the organization and delivery of care and can be especially useful in uncovering what really happens in particular healthcare scenarios [13].

Thus, the aim of this study is to evaluate the dispensing practice of antibiotics in community pharmacy settings and compare between the different patterns of drug dispensing (with prescription, self-medication, and recommendation of pharmacist) in terms of the appropriateness of drug to the indication, dosage, and duration of antibiotics dispensed to patients.

## Methodology

The study was a cross sectional observational study. The study protocol was approved by the Institutional Review Board of ACDIMA biocenter, Amman, Jordan (protocol number AB-1-16). Research assistants (n = 7) were distributed in seven community pharmacies in four cities in Jordan (Amman, Irbid, Zarqa and Madaba). The research assistants included six Doctor of Pharmacy (PharmD) students in their sixth year (final year) of studies. They were trained on the method in general, filling of the data collection form, rules of approaching the customers and communication skills. They also consented to the ethical code designed for this study in order to maintain the anonymity of participating individuals and to protect the integrity of the data obtained.

Recruitment of pharmacies was made by personal contact and obtaining verbal consent of the pharmacist in charge. Verbal consent was obtained because several pharmacists-in-charge indicated that they were more comfortable in providing verbal consent rather than a written one. The reason given was that the latter might have some element of risk in breaching the anonymity of the pharmacist, especially where that the study revealed any potential malpractice. This approach was approved by the IRB. According to Jordanian legislation, pharmacies are run by licensed pharmacists who hold a BSc degree in pharmacy or PharmD. It also requires that there must be a qualified pharmacist on duty during all opening hours. The pharmacists can be supported by pharmacy assistants who hold a diploma degree in pharmacy a (two year programme in middle college). According to Jordanian pharmacy law, each pharmacy must have a licensed pharmacist in charge (registered in Ministry of Health). To obtain an approval to conduct the study in a pharmacy, the permission of the pharmacist in charge was sought. Based on his/her consent, the other workers/dispensers provided their consent. During recruitment, two pharmacists in charge refused to participate, so their pharmacies were not included in the study. However, seven pharmacists in charge agreed to participate and provided their consent. Accordingly, 12 pharmacists in these seven pharmacies participated in the study. Demographic information of participating pharmacists/pharmacy assistants such as age, degree, country of graduation, and years of experience were collected (S1 Appendix).

Research assistants were requested to observe all patient-pharmacist interactions that involved antibiotics whether by prescription or not. When antibiotic dispensing to the customer was conducted, the researcher intervened to collect the necessary information from the customer and the prescription (if present) after getting a verbal consent from the customer to participate in the study. The customers were told that they have the right to withdraw from the study and if they refuse to participate, the service that they receive in the pharmacy will not be affected. The information collected from the customers was immediately recorded in a data collection form (S2 Appendix). The identity of the participants was anonymized. The form included information about the customer and the patient such as age, gender, education, to whom the drug is dispensed, diagnosis (in case of prescription) or symptoms and complaint (in case of nonprescription medication), others such as (comorbidities, chronic diseases, allergies and pregnancy), antibiotics dispensed (trade name, active ingredient, strength), dosage, duration of treatment, cost, and the dispensing practice whether by prescription, direct self-medication (i.e., requesting a specific drug by name or description), or indirect self-medication (i.e., presenting symptoms to the pharmacist). The collection of data using these data collection forms was validated by conducting a pilot study in four pharmacies for 50 cases of antibiotic dispensing. These cases were used to enhance the training of the research assistants and improve the form. The data collection form was reviewed by two PhD holders in clinical and pharmacy practice to ensure face validity, whereas content validity was assured by extensive review of the literature [14–16]. Antibiotic dispensing practice was evaluated over a period of four months from February 2016 to May 2016. The collection of data was performed by research assistants three days per week on average. The appropriateness of the dosage and duration were assessed versus the written diagnosis or presented symptoms. Similarly, the appropriateness of the antibiotic indication was assessed in terms of the suitability of the antibiotic to the infection, the age of the patient, the comorbidities (e.g. chronic diseases or allergies) and pregnancy. The assessment was made based on the empirical treatment suggested by the references Lexicomp (2017), UptoDate (2017), the manufacturer's recommendation and the agreement of the expert panel within the research team [17,18]. These references were selected since they contain summaries of other guidelines and there are no national or local guidelines to cite. For antibiotics administered to infants or children where the dosage was calculated based on body weight, World Health Organization charts were consulted [19,20], and the median weight (50^th^ percentile) was considered for the calculation of the dose.

## Statistical analysis

Data were analyzed using SPSS software version 22. Descriptive data were expressed as frequencies and percentages. The Shapiro–Wilk test was used to test for normality and if p-value was >0.05 then data was considered normally distributed. The chi-square test was used to test significant differences between categorical variables. The t-test was used to test significant differences between continuous variables. All p-values were two-sided and any p-values less than 0.05 were considered statistically significant. Factors which were shown to have significant effect on wrong dose or duration of antibiotic dispensing (p<0.05) were subjected to multivariate analysis using binary logistic regression to evaluate the potential predictors of inappropriate dispensing of antibiotic.

## Results

### Characteristics of antibiotic dispensers and patients/customers

Dispensing practices related to antibiotics were assessed over a period of four months. During this period, 434 customers/patients consented to participate in the study and nine customers/patients refused to provide their consent. Accordingly, 434 antibiotic encounters were assessed. Regarding the dispensing staff, 12 pharmacists working in seven pharmacies in four cities in Jordan participated in the study. The majority (43.3%) was in the age range of 25–30 years. About half of the patients were male (50.2%). More than half of the patients (53.7%) for whom antibiotics were requested were in the age range 19–45 years. Characteristics of staff and patients/ customers are summarized in Table 1 and Table 2 respectively.

**Table 1:** Demographic characteristics of dispensing staff (N = 12). Number of pharmacies participated in the study (N = 7). Total antibiotic interactions were 434.

Dispensing Staff Characteristic	Number of staff (N = 12); N (%)	Frequency of Interactions; (N = 434); N (%)
**Age (years)**		
19-<25	3 (25)	20 (4.6)
25-<31	6 (50)	188 (43.3)
31–40	1 (8.3)	100 (23.0)
>40	2 (16.7)	126 (29.0)
**Gender**		
Females	9 (75)	231 (90.2)
Males	3 (25)	25 (9.8)
**Years of Experience**		
<1 (Trainee)	1 (8.3)	6 (1.4)
1–2	2(16.7)	14 (3.2)
3–5	3 (25)	181 (41.7)
6–10	4 (33.3)	107 (24.7)
>10	2 (16.7)	126 (29.0)
**Country of graduation**		
Jordan	11 (91.7)	402 (92.6)
Others*	1 (0.3)	32 (7.4)
**Degree of study**		
BSc	8 (66.7)	272 (62.7)
Diploma	3 (25)	156 (35.9)
Trainee	1 (8.3)	6 (1.4)
**Pharmacy location**	**No. of pharmacies N = 7; N (%)**	
Western Amman	2 (28.6)	29 (6.7)
Eastern Amman	1.(14.3)	115 (26.5)
Zarqa	1 (14.3)	124 (28.6)
Madaba	1 (14.3)	102 (23.5)
Irbid	2 (28.6)	64 (14.7)

* Pakistan

**Table 2:** Demographic characteristics of patients/customers (N = 434 antibiotic dispensing cases).

Patient/Customer Characteristics	AB Dispensing Cases, N (%)
**To whom prescription is dispensed**	
Patient him/her-self	206 (47.8)
Others (Relative/friend)	225 (52.2)
**Age of patient (years)**	
<1	8 (1.9)
1<6	72 (16.8)
6<12	47 (11.0)
12<19	28 (6.5)
19–45	230 (53.7)
>45	43 (10.1)
**Patient gender**	
Female	214 (49.5)
Male	218 (50.5)
**Customer education**	
Post graduate	5 (1.2)
BSc	204 (49.5)
Diploma	40 (9.7)
High School	84 (20.4)
School	64 (15.5)
Others*	15 (3.6)

* Not educated or refused to declare

### Practice of antibiotic dispensing

**Fig 1:**
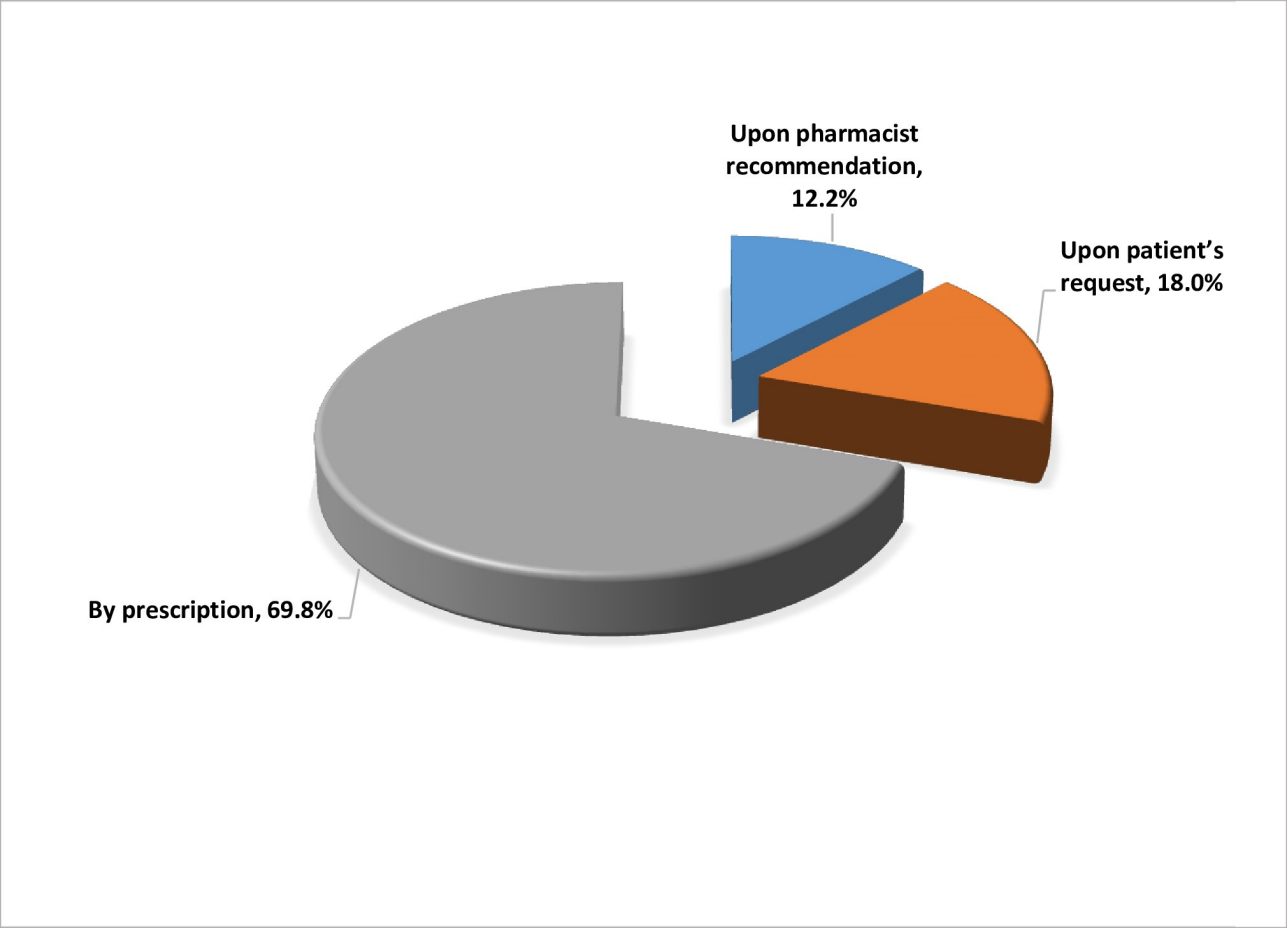
Dispensing pattern of antibiotics in Jordan.

In this study, 457 antibiotics were dispensed with a total cost of JOD 3,570 ($ 5,028). The majority of antibiotics dispensed were oral drugs (87.6%) followed by topical/local (8.5%) and injectable (3.9%) antibiotics. More than two thirds (69.8%) of the dispensed antibiotics were prescribed by physicians or dentists (Fig 1). Generic brands of antibiotics dispensed comprised 67% of the dispensed antibiotics. The characteristics of antibiotic dispensing practices in pharmacies are described in Table 3.

**Table 3:** Description of antibiotic dispensing practice in community pharmacies. N = 434 antibiotic interactions.

**Antibiotic Request Type**	**Total requests = 434; N(%)**
Prescription	303 (69.8)
Non prescription	
• Direct self-medication by name and dose	58 (13.4)
• Direct self-medication by description	20 (4.6)
• Indirect self-medication by description of symptoms	53 (12.2)
**Antibiotic Brand, N (%)**	**Total interactions = 434**
• Generic	289 (66.6)
• Originator	145 (32.6)
Appropriateness of Dose,* N (%)	**Total interactions evaluated = 428**
• Correct	197 (46.9)
• Low dose	65 (15.5)
• High dose	77 (18.3)
• Not Indicated	52 (12.4)
• Only specific cases	29 (6.9)
• No dose given to patient	8 (1.8)
**Duration of antibiotic treatment (days), Median (Range)**	**5 (1–120)**
Appropriateness of antibiotic duration*	**Total interactions evaluated = 428**
• Correct	175 (40.9)
• Shorter duration	146 (34.1)
• Long duration	25 (5.8)
• Not indicated	52 (12.2)
• Only in specific cases	17 (4.0)
• No duration given to patient	13 (3.0)
**Cost of antibiotic dispensed (JD), Median (Range)**	6 (1–46)

*: based on Lexicomp 2017, UptoDate.

JD: Jordan Dinar

About one third (30.2%) of the dispensed antibiotics were without prescriptions (Fig 1). Antibiotics dispensed without a prescription were oral or topical, none were an injectable. Dispensing of nonprescription antibiotics was obvious in Madaba compared to other cities (43.0%, p < 0.00). It was also higher among customers with BSc degrees (59.5%, p < 0.005; Table 2).

**Fig 2:**
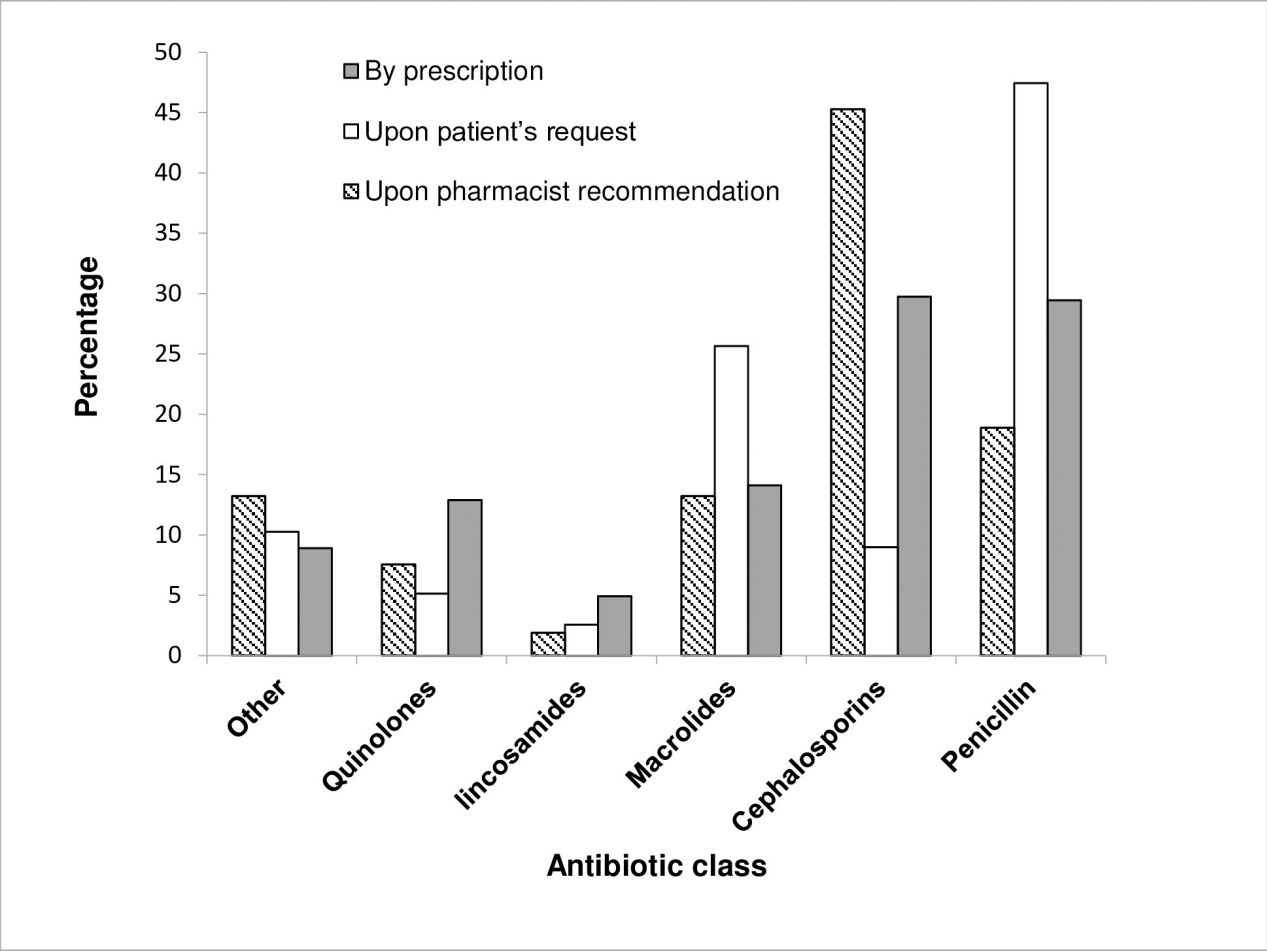
The most common classes of antibiotics dispensed by prescription, upon patient’s request, or upon pharmacist's recommendation.

Fig 2 shows the main classes of antibiotics dispensed in the participating pharmacies. Penicillins and cephalosporins (first to third generations) were the most common antibiotics dispensed by prescription. Penicillins were also the most common antibiotics requested by patients. However, among all antibiotic classes, cephalosporins were the most commonly recommended and dispensed antibiotics by the pharmacists. It is noteworthy to mention that third generation cephalosporins were the highest cephalosporins to be dispensed in all categories (prescriptions, self-medication, or pharmacist's recommendation). In general, the top three antibiotics dispensed were amoxicillin-clavulanate (24.2%), cefixime (11.3%), and azithromycin (10.1%). Amoxicillin-clavulanate was the most commonly dispensed by those with 3–5 years of experience (32.4%, *p* = 0.008) for patients in the age range 6–12 years (42.6%, *p* = 0.003). Amoxicillin-clavulanate was dispensed in its generic brands (87.6%, *p* < 0.000), mainly in Eastern Amman and Zarqa (33% and 33.9%, respectively, *p* = 0.01).

**Fig 3:**
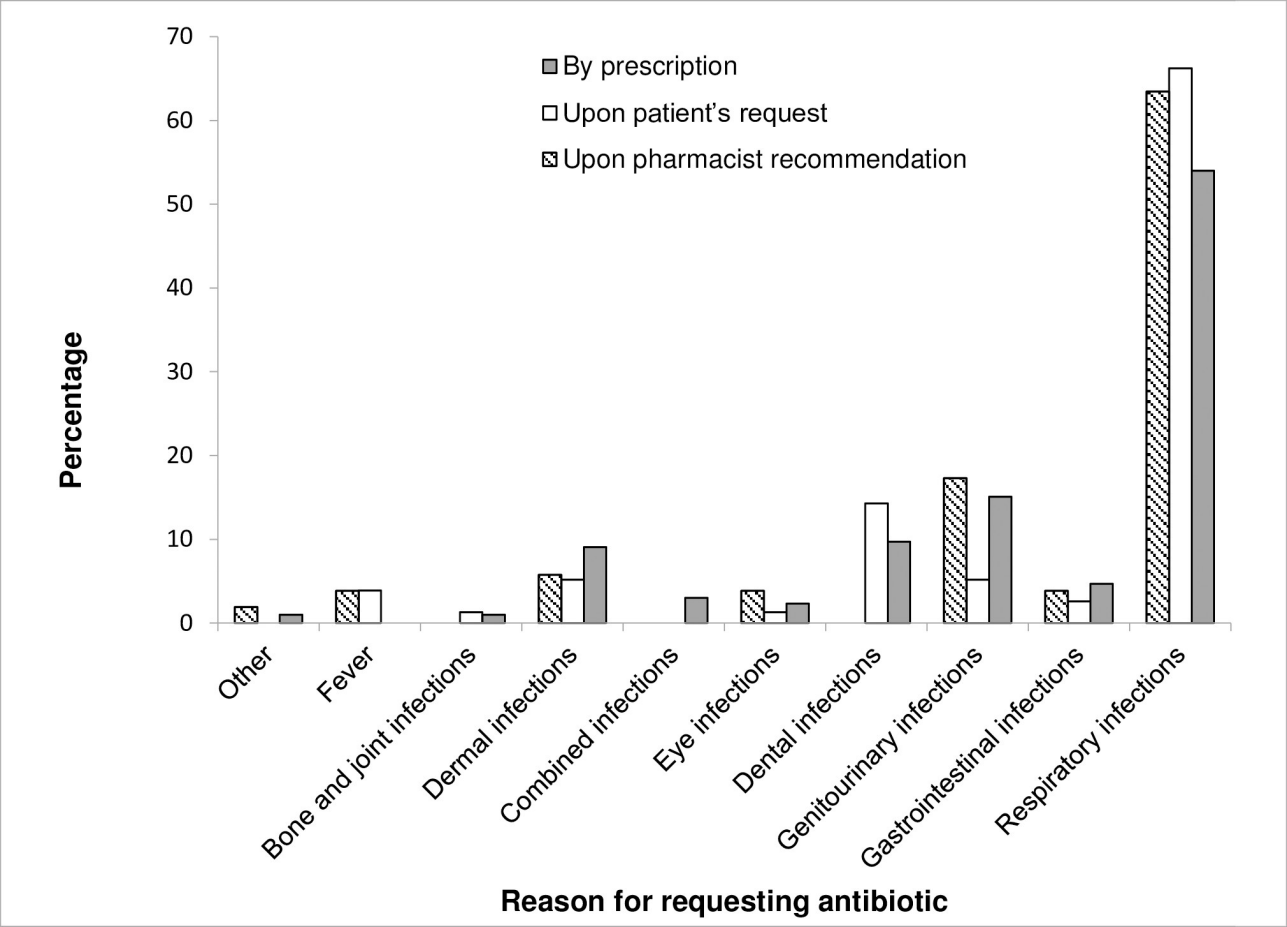
The types of infections to which the patients procured antibiotics, based on prescription, their request, or upon pharmacist's recommendation.

The most common reasons for requesting antibiotics were respiratory tract infections (59.5%). This was obvious for the three categories of antibiotic dispensing: prescription (57%), self-medication (66%), and pharmacist recommendation (63.5%; Fig 3). Of the antibiotics dispensed without prescription to treat respiratory tract infections, 16 out of 84 antibiotics (19%) were dispensed to patients who presented with cold or flu symptoms. Nevertheless, only 3.7% of the antibiotics prescribed for respiratory tract infections were for diagnosed flu or cold. However, the diagnosis was rarely written in the prescription and only reported by patients upon asking.

**Fig 4:**
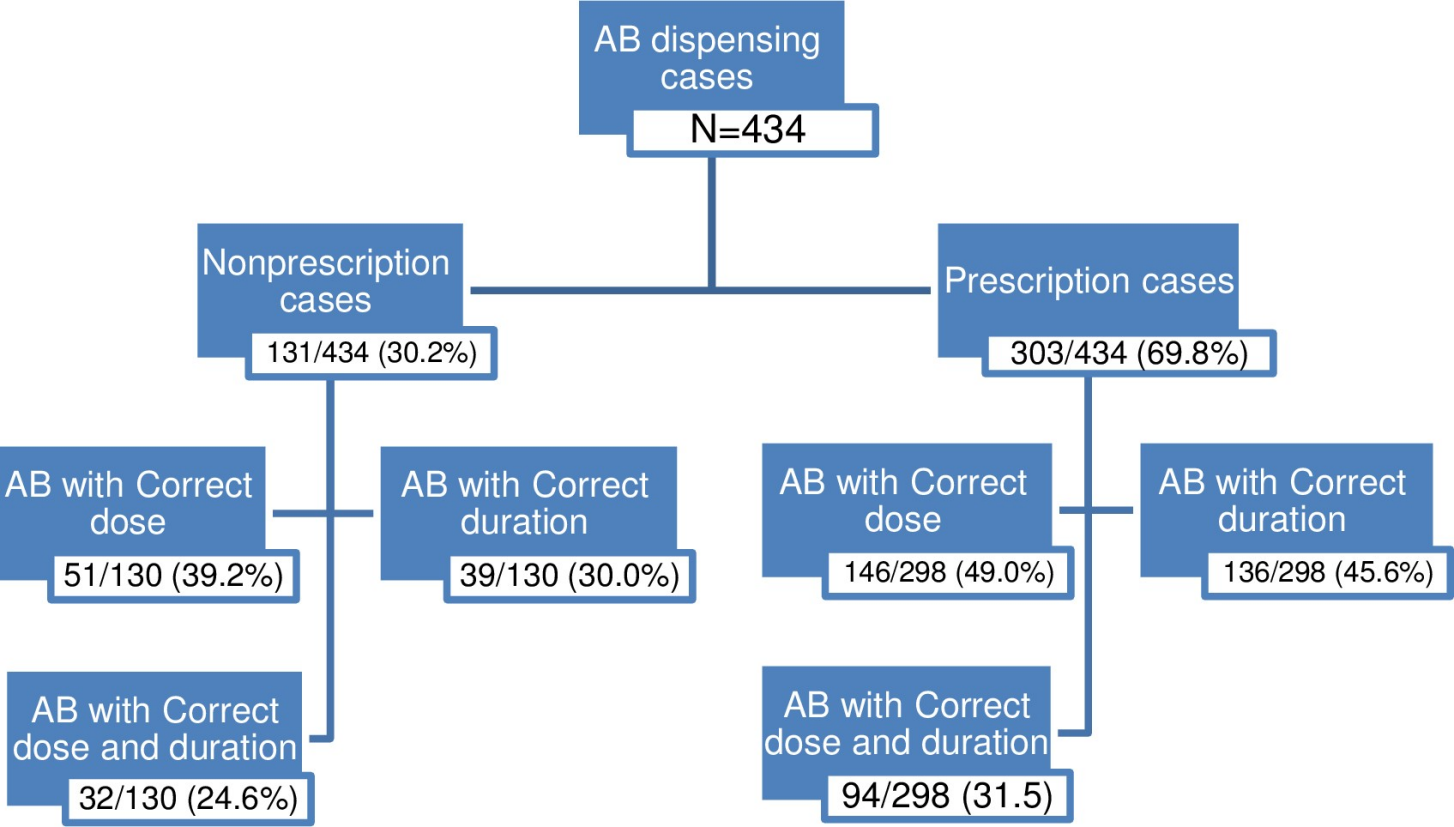
Frequencies of antibiotic (AB) dispensing cases, prescription and nonprescription, in terms of appropriateness of AB dose and duration.

The appropriateness of the dosage and duration of the dispensed antibiotics were evaluated based on the adopted references of this study [17,18]. Of the antibiotics dispensed by prescription, 31.5% were the correct dosage and duration as recommended in the references, compared to 24.6% of the antibiotics dispensed without prescription (*p*-value = 0.002; Fig 4). Azithromycin was dispensed at higher dosages and shorter durations than recommended in the references in 56.6% and 59.1% of the cases, respectively. Cefuroxime was dispensed at higher dosages and shorter durations than recommended in the references in 45% and 75% of the cases, respectively. On the other hand, clindamycin was dispensed at lower dosages and shorter durations than recommended in references in one third of the cases. The most common antibiotic deemed inappropriate for indication was lincomycin (43.8%).

Only 26.1% of ciprofloxacin and 23.8% of amoxicillin-clavulanate were dispensed with correct dosages and durations according to the references.

The demographic characteristics of the pharmacists (e.g. age, gender or years of experience) were found not to influence the degree of appropriate dosage and duration of antibiotics dispensed to patients without prescription. Univariate analysis showed that years of experience of the dispenser (>10 years) and type of antibiotic request (self-medication) were associated with inappropriate antibiotic dispensing (p-value <0.05). These variables were subjected to logistic regression analysis (backward LR). The analysis indicated that antibiotics dispensed via self-medication were significantly and independently associated with inappropriate antibiotic dispensing (Odds ratio 2.07, 95% CI 1.37–3.15, p = 0.001).

Of the 434 dispensed antibiotic cases, 23 cases had two antibiotics dispensed together to treat certain infections. The most common combination was ceftriaxone injection with an oral beta lactam (9/23). All the cases of two antibiotics dispensed together were prescribed by physicians.

## Discussion

Misuse and overuse of antibiotics are worldwide problems that contribute substantially to the emergence and flaring up of antimicrobial resistance. This study is the first observational study that aimed to evaluate the dispensing practice of antibiotics in community pharmacy settings and compare between the different patterns of drug dispensing in terms of the appropriateness of drug, dosage, and duration of antibiotics dispensed to patients.

Legislation in Jordan requires that prescription drugs are only dispensed by licensed pharmacists. Additionally, Jordanian Food and Drug Administration (JFDA) regulations require that antibiotics should not be dispensed without prescription. However, these regulations are not fully enforced. In Jordan, pharmacy assistants and sometimes trainees dispense prescription drugs to customers. This breach of Jordanian national legislation was observed in this study. Out of the 12 dispensers, three pharmacy assistants and one trainee did 37% and 6% antibiotic interactions respectively in this study.

In the current study, around one third of the dispensed antibiotics were without prescription. They were either based on pharmacist recommendation or directly requested by the patient. This is in concordance with previous studies worldwide [21–24]. In these studies, low income and lack of health insurance were among the main factors that contributed to self-medication. In fact, these factors also apply to Jordan. Over the years, including the last three years, the World Bank has classified Jordan within the middle-income class (ranging from lower middle to upper middle class) [25]. Therefore, this low income could drive the individuals to seek self-medication rather than medical treatment. On the other hand, health insurance in Jordan covers around 55% of the overall population (Jordanians and non-Jordanians) [26]. The majority of Jordanians are covered by the Ministry of Health or Royal Medical services insurance where patients get their medications from the institutions of these sectors [26]. Nevertheless, a considerable proportion of patients is still uncovered by health insurance and gets their medications from community pharmacies, where the study was conducted. This would explain the high proportion of non-prescription antibiotics dispensed. In a study in Egypt, nonprescription antibiotics comprised 27% of the dispensed antibiotics [16]. In Pakistan, a lower middle income country [27], 50% of non-medical university students declared self-medicating themselves with antibiotics [24]. Even in Europe, self-medication was reported. In Northern and Western Europe, in which people have higher income, the rates of self-medication were in the range of 0.1%-0.9% while in Southern and Eastern Europe the rates were much higher ranging from 0.7% -20% [22]. A study in Mexico, showed that the lack of government insurance increased the tendency to self-medication [23]. Similarly, a large study in rural India performed on more than 21,000 individuals demonstrated that insuring people resulted in reducing their self-medication. Also, there was a relationship between the length of the insurance period and the tendency of people to seek medical help rather than self-medicate themselves [21]. In our study, the results of the logistic regression analysis show that requesting an antibiotic for self-medication increases the risk of receiving inappropriate antibiotic dispensing by 2.07 times. Other studies [4,8] have shown that self-medication is one of the major causes linked to the increase in antimicrobial resistance and one of the main challenges that should be addressed by authorities to tackle the problem of resistance.

In the present study, beta-lactam antibiotics (penicillins and cephalosporins) were the most frequently dispensed antibiotics whether for prescription or nonprescription antibiotics (59%). In particular, amoxicillin or amoxicillin-clavulanate comprised about 24.2% of all antibiotics dispensed. This could be due to the fact that the most common infections reported in this study were upper respiratory tract infections (URTI) followed by genitourinary tract infections. The empirical treatment for the majority of these infections is beta lactams [18]. Besides, these antibiotics are generally safe (with low side effects) and encourage the pharmacists to recommend them. Moreover, since these antibiotics are widely prescribed [16,22], most people are familiar with these medications and recommend them to relatives and friends [7,22,28]. Similar results were obtained in other studies performed in different parts of the world [16,22]. In Europe, the most frequently used antibiotics for self-medication were penicillins to treat URTI and urinary tract infections (UTI) [22]. In a study in Egypt, the most widely dispensed antibiotics were penicillins and cephalosporins followed by flouroquinolones. The major infections to which antibiotics were dispensed were flu, sore throat, and UTI [16].

Some antibiotics were prescribed for conditions for which they were not indicated or their indication should be saved for only specific cases. This was obvious in lincomycin which was prescribed in many cases although its use should be reserved for highly resistant bacteria not responding to other treatments. The main indications for lincomycin prescription were dental issues (pulpitis or prophylaxis before dental surgery) or for URTI (tonsillitis, pharyngitis). Such cases could have many other better alternatives than lincomycin [17,18]. Some antibiotics were prescribed to treat viral infections such as the common cold and influenza (3.7% of prescribed antibiotics compared to 19% of those dispensed without prescription). However, the fear from secondary bacterial infections for some patients may explain their prescribing.

Another observation made was the tendency of physicians to prescribe the higher generations of different drug classes. For example, third generation cephalosporins comprised 68% of the total cephalosporins prescribed to treat patients suffering from URTI or UTI. Similarly, more than half of the fluoroquinolones prescribed were from the third and fourth generations. These were prescribed to adult patients suffering from URTI or UTI. However, all treatment guidelines recommend saving higher generation antibiotics for resistant cases. Part of this trend among physicians can be explained by the strong marketing promotions of new higher generation antibiotics. Also, probably to some extent, there is a lack of awareness about the seriousness of antibiotic resistance due to absence of mandatory continuous education programs. Such programs are supposed to keep the physicians updated with the latest news about microbial resistance and the best ways to tackle them [29].

Of the antibiotics dispensed 67% were generics. This is not uncommon in a country with middle income like Jordan. Therefore, the patients tend to buy the generics which have lower prices than the originators and the physicians tend to prescribe generics for patients mainly if they do not have health insurance. In addition, the low income of the population affected the manner of getting treatment.

The striking results obtained from this study were the relatively low number of appropriate dosages or durations or both for the dispensed antibiotics whether by prescription or per pharmacist recommendation. The appropriateness of dosage and duration were judged based on the references used in this study (Lexicomp, 2017; UpToDate 2017, and manufacturer's insert) [17,18]. Only around 30% of the antibiotics dispensed by prescription were correct in terms of both dosage and duration. However, this disappointing finding could have been overestimated due to the limitations of the study.

Among antibiotics prescribed by physician, the most common deviances from guideline recommendations included azithromycin and cefuroxime. Azithromycin was prescribed for adults at 500 mg/day for 3 days for the majority of infections encountered. Based on the references used in this study, the infections reported required treatment with 250 mg/day for 5 days. Cefuroxime was prescribed at 500 mg twice daily for UTI instead of 250 mg twice daily.

The dispensing of antibiotics without a prescription is a worldwide problem, despite that in most countries the pharmacist is breaching the law in this act. Among the motivations for pharmacists to dispense nonprescription antibiotics were pressure from the customers, to prevent customers to seek counselling from other pharmacies, lack of education and lack of legal enforcement [16,30]. In the current study, the dispensed nonprescription antibiotics comprised around 30%. These were dispensed either according to pharmacist recommendation or based on the request of the customer/patient by giving the name or the description of the drug. The infections treated by these antibiotics included respiratory, genitourinary, dental, dermal, fever, and others. The most common antibiotics dispensed were amoxicillin or amoxicillin-clavulanate, cephalosporins (first, second, and third generations), macrolides, flouroquinolones (second, third, and fourth generations), lincosamides, and to a lesser extent other antibiotics. Broad spectrum antibiotics such as cefixime, cefpodoxime, levofloxacin, and moxifloxacin can be easily obtained from pharmacies. Even antibiotics that have serious side effects such as lincomycin were bought from pharmacies without prescription. These results are disturbing since they indicate the severity of antibiotic misuse in Jordan. Hence, this may justify to some extent the dissemination of antibiotic resistance in Jordan. In the last years, multiple drug resistant bacteria have been increasingly isolated from different sources in Jordan. They were isolated from patients, surface and ground raw water, green vegetables, and even from household settings [31–33]. Although the Jordanian Food and Drug Administration (JFDA) requested that all antibiotic packs should have a statement indicating that antibiotics should be dispensed by prescription only, no action has been taken by the health authorities to prevent their over-the-counter sale.

Of the non-prescribed antibiotic cases only 24.6% were in the correct dosages and durations. Compared to prescribed antibiotics, the appropriateness of the dosages and durations of antibiotics recommended by pharmacists was significantly lower (31.5%). This stresses the importance of enforcing the dispensing of antibiotics with prescription only to ensure an appropriate dosage regimen given to patients. In addition, more efforts should be directed to educate the physicians on following the principle guidelines for the rational use of antibiotics.

In this study, we were able to identify three elements of inappropriate pharmacy practice or irrational use of antibiotics. These elements are (i) the consumer or patient who is seeking self-medication through buying antibiotics over the counter; (ii) the pharmacist by dispensing antibiotics to patients without prescription and/ or dispensing antibiotics in inappropriate dose, duration or indication and (iii) the physician through prescribing antibiotics with inappropriate dose, duration or indication. The JFDA requires that all antibiotics dispensed by trained pharmacists must have a statement indicating that the antibiotics should be dispensed by prescription only. Whilst this requirement is not legislative it is a requirement of obtaining a license for the dispensing of each individual antibiotic. Several studies conducted at national or international levels worldwide [4,34,35], have identified the misuse and irrational use of antibiotics among the factors contributing to antimicrobial resistance worldwide. These studies urge the appropriate authorities to take actions to combat the antimicrobial resistance crisis facing the world [2–4,34–36].

## Limitations of the study

The findings of the study should be interpreted with the following limitations in mind:

- The study was conducted in only seven pharmacies in four cities in Jordan; hence, the results might not be generalizable. However, as the first study of its kind in Jordan, and bearing in mind the observational type of the study, it is considered satisfactory to provide background data at this stage.
- The evaluation of the appropriateness of the dosages and durations for the prescribed antibiotics were judged based on the empirical treatment and the diagnosis only without knowing the history of the patient or laboratory results that drove the physician to prescribe a certain drug with such a dosage and duration.
- Although every effort had been made to assure pharmacists of the confidentiality and anonymity of the study and that it is done solely for research purposes by an academic team, the Hawthorne effect could not be totally negated, as some pharmacists may have behaved differently in the presence of the researchers, which may have affected the results.
- The recruitment method may have carried some sort of selection bias, as only interested pharmacists were included. Nevertheless, the researchers believe that pharmacy settings in Jordan are very similar.

## Conclusion

The dispensing of nonprescription antibiotics comprises a substantial proportion of antibiotics dispensed in community pharmacies in Jordan. In addition, a significant proportion of antibiotics are dispensed inappropriately either in terms of choice, dose, or duration. This will lead to the emergence of multiple drug resistant microbes. The lack of enforced regulations in Jordan is encouraging pharmacists to violate the regulations that govern the appropriate and safe dispensing of antibiotics. Moreover, there is a real need to implement continuous education programs to physicians and pharmacists to reveal to them the latest situation of antibiotic resistance and the most rational use of antibiotics.

## Supporting information

S1 Appendix Data collection form from dispensers. (DOCX)

S2 Appendix Data collection form from customers/patients. (DOCX)

S1 Table All relevant raw data collected from the dispensers and customers/patients. (XLSX)
