# Supplementary material for: Evaluation of antibiotic dispensing practice in community pharmacies in Jordan: A cross sectional study
Source: PLoS One. 2019 Apr 29;14(4):e0216115. doi: 10.1371/journal.pone.0216115 (PMC6488076; doi:10.1371/journal.pone.0216115)
Supplement: S1 Appendix — (DOCX) [file pone.0216115.s001.docx]

**S1 Appendix**

**“Prevalence of Antibiotic Self medication in Community Pharmacy Setting in Jordan”**

**Data Collection Form/Dispenser**

To be filled once for the staff consented to participate in the study

**Pharmacy location (City):**

**Demographic Details about the Dispenser of service**

| Dispenser initials | Gender | Degree (e.g BSC, Pharm D, Diploma, trainee, etc) | Age (yr) | Years of experience | Country of graduation |
| --- | --- | --- | --- | --- | --- |
|  |  |  |  |  |  |
|  |  |  |  |  |  |
|  |  |  |  |  |  |
|  |  |  |  |  |  |
|  |  |  |  |  |  |
