# Supplementary material for: Evaluation of antibiotic dispensing practice in community pharmacies in Jordan: A cross sectional study
Source: PLoS One. 2019 Apr 29;14(4):e0216115. doi: 10.1371/journal.pone.0216115 (PMC6488076; doi:10.1371/journal.pone.0216115)
Supplement: S2 Appendix — (DOCX) [file pone.0216115.s002.docx]

**S2 Appendix**

**“Prevalence of Antibiotic Self medication in Community Pharmacy Setting in Jordan”**

**Data Collection form/Customer or patient**

(to be filled for each customer/patient)

**Date:………………….. Research Assistant name:……………………**

**Patient's age…………….. Gender………………. Dispenser initials:…………………….**

| **Part A: Details of the patient** | | | | **Part B: Details of Interaction** | | | | | |
| --- | --- | --- | --- | --- | --- | --- | --- | --- | --- |
| Diagnosis (for prescription AB)  Symptoms/Complaint (for non-prescription AB) | Others: comorbidities/chronic diseases/ Allergies/ pregnancy | To whom?  (Him/her-self /others (specify)) | Customer education | Request Type* | Antibiotic Trade name/  dosage form / Strength | Active ingredient | Dosage given | Duration of treatment given | Cost of ABs |
|  |  |  |  |  |  |  |  |  |  |

* Request codes: **1**: On Prescription; **2a**: Direct self medication by name and/or dose; **2b**: Direct self medication by description ; **3**: Indirect self medication (ie- presenting symptoms and asking for advice)

Page no.:
